# Cu^2+^-Chelating Mesoporous Silica Nanoparticles for Synergistic Chemotherapy/Chemodynamic Therapy

**DOI:** 10.3390/pharmaceutics14061200

**Published:** 2022-06-04

**Authors:** Yanyan Zhang, Jiadong Lou, Gareth R. Williams, Yuhan Ye, Dandan Ren, Anhua Shi, Junzi Wu, Wenling Chen, Li-Min Zhu

**Affiliations:** 1 College of Chemistry, Chemical Engineering and Biotechnology, Donghua University, Shanghai 201620, China; zhangyy518@163.com (Y.Z.); loujiadong666@163.com (J.L.); yeyuhan122@sina.com (Y.Y.); 19821273681@163.com (D.R.); 2 UCL School of Pharmacy, University College London, 29-39 Brunswick Square, London WC1N 1AX, UK; g.williams@ucl.ac.uk; 3 The Key Laboratory of Microcosmic Syndrome Differentiation, Education Department of Yunnan, Yunnan University of Chinese Medicine, Kunming 650500, China; ynshianhua@126.com (A.S.); xnfz@ynutcm.edu.cn (J.W.); 4 School of Clinical Medicine, Yunnan University of Chinese Medicine, Kunming 650500, China

**Keywords:** mesoporous silica nanoparticles, chelation, gatekeeper, synergistic treatment

## Abstract

In this study, a pH-responsive controlled-release mesoporous silica nanoparticle (MSN) formulation was developed. The MSNs were functionalized with a histidine (His)-tagged targeting peptide (B3int) through an amide bond, and loaded with an anticancer drug (cisplatin (CP)) and a lysosomal destabilization mediator (chloroquine (CQ)). Cu^2+^ was then used to seal the pores of the MSNs via chelation with the His-tag. The resultant nanoparticles showed pH-responsive drug release, and could effectively target tumor cells via the targeting effect of B3int. The presence of CP and Cu^2+^ permits reactive oxygen species to be generated inside cells; thus, the chemotherapeutic effect of CP is augmented by chemodynamic therapy. In vitro and in vivo experiments showed that the nanoparticles are able to effectively kill tumor cells. An in vivo cancer model revealed that the nanoparticles increase apoptosis in tumor cells, and thereby diminish the tumor volume. No off-target toxicity was noted. It thus appears that the functionalized MSNs developed in this work have great potential for targeted, synergistic anticancer therapies.

## 1. Introduction

The use of nanoparticles to deliver chemotherapeutic drugs to tumor tissue has become a hot topic in cancer therapy research. Mesoporous silica nanoparticles (MSNs) have been widely used to this end because of their large specific surface area, easy-to-adjust pore size, surface functionalization ability, and biocompatibility, among other properties [1,2,3]. However, the mesoporous nature of the material predisposes MSNs to undergo rapid release at non-target sites. The question of how to avoid drug release before the formulation reaches the tumor tissues is still a challenge [4], which is often overcome by sealing or “capping” the surface pores. Since MSNs were first sealed with coumarin in 2003 [5], various “gatekeepers” to cap the surface pores have been developed. These can comprise, for instance, proteins [6,7], DNA [8], polymer molecules [9,10], prodrugs [11], or nanoparticles [12,13]. Clever design of the system means that the gatekeepers can be triggered and uncapped by stimuli specific to the tumor microenvironment (TME) (e.g., pH [14], redox [15], enzymes [16], etc.) or by the application of external stimuli (e.g., light [17], temperature [18], magnetic field [19], etc.). For instance, previous work in our group used MoS_2_ nanosheets to cap the exterior mesopores of periodic mesoporous silicone nanoparticles using electrostatic interactions and mercaptan reactions [20]. In other work, Cheng et al. linked a hydrophobic alkyl chain to a hydrophilic peptide chain and grafted it onto the surface of MSNs to achieve self-sealing pores via hydrophobic interactions [21]. In further study, Lee et al. capped MSNs with peptide gatekeepers containing disulfide bonds, which could release drugs via stimulus-responsive conformational transition [22]. 

There are often shortcomings when treating cancer using a single therapy, as a result of which synergistic, multicomponent, strategies have received increasing attention [23,24,25,26]. The combination of chemodynamic therapy (CDT) and chemotherapy is particularly powerful [27,28,29]. Cu^2+^ is widely used in CDT because it can produce cytotoxic reactive oxygen species (ROS) via Fenton-like reactions with the high concentrations of H_2_O_2_ typically found inside tumor cells. At the same time, excessive glutathione (GSH) in the TME can be consumed by Cu^2+^, preserving the ·OH radicals produced by CDT [30]. Cisplatin (CP) is a chemotherapeutic drug widely used in clinical cancer treatment [31]. It can enhance the activity of nicotinamide adenine dinucleotide phosphate oxidase and produce H_2_O_2_ with the assistance of superoxide dismutase [32,33,34]. Thus, CP also has CDT potential. However, platinum-based drugs are easily inactivated by intracellular reducing agents (e.g., GSH) [35,36]. Hence, the addition of Cu^2+^ can help to protect both the chemotherapeutic and CDT activity of drugs such as CP [37].

The use of Cu^2+^ in CDT can also offer the opportunity for some degree of passive targeting. Cu^2+^ can be chelated with a range of pH-sensitive moieties—for instance, the histidine tag (His-tag) [38,39]. In an acidic environment (pH 4~5) such as that found in the lysosome, the His is protonated and, thus, the strength of coordinate bonds with Cu^2+^ is weakened, and the complex falls apart [40]. However, passive targeting is often insufficient, and in order to achieve precise delivery of drug-loaded nanoparticles to tumors, carriers generally need to be modified with targeting groups. Integrin β3 is a cell-surface adhesion factor involved in the occurrence and development of tumors, and is overexpressed on the surface of a variety of cancer cells [41,42,43,44]. Therefore, ligands to integrin β3 can be employed as nanoparticle modifiers to target therapy. The B3int peptide is one such ligand [45], with high affinity for integrin β3. This can aid the entry of the drug into the cytosol, but it will typically be trafficked into lysozymes. Drug retention in the lysosome can be problematic, but species such as chloroquine (CQ) can be used to overcome this issue. CQ causes lysosomal membrane permeability, leading to drug escape [46].

**Scheme 1:**
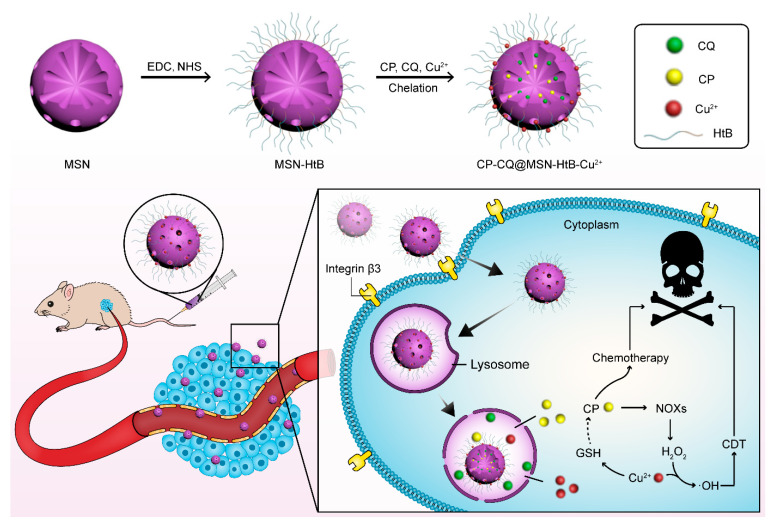
Construction of the CP/CQ@MSN−HtB/Cu^2+^ nanoplatform for synergistic chemotherapy/chemodynamic therapy.

In this study, we developed MSNs with surface pores sealed by the chelation of Cu^2+^ and a His-tag (Scheme 1). The HtB peptide (His-tag-B3int) was first attached to the surface of the MSNs through an amide bond. After loading CP and CQ, the nanoparticles were sealed by the chelation of Cu^2+^ with the His imidazole ring. The resultant CP/CQ@MSN−HtB/Cu^2+^ nanoparticles were fully characterized, and their tumor-targeting and -killing abilities were explored both in vitro and in vivo.

## 2. Materials and Methods

Tetraethyl orthosilicate (TEOS), copper chloride (CuCl_2_), and triethanolamine (TEA) were purchased from Sinopharm Chemical Reagent Co. Ltd. (Shanghai, China). Hexadecyl trimethyl ammonium bromide (CTAB), o-phenylenediamine (OPD), dimethyl sulfoxide (DMSO), cisplatin (CP), chloroquine (CQ), and doxorubicin (DOX) were provided by Macklin Biochemical Technology Co. Ltd. (Shanghai, China). 3-Aminopropyltriethoxysilane (APTES), methylene blue (MB), 1-(3-dimethylaminopropyl)-3-ethylcarbodiimide hydrochloride (EDC), N-hydroxysuccinimide (NHS), and pyrocatechol violet (PV) were acquired from Aladdin Biochemical Technology Co. Ltd. (Shanghai, China). The HtB peptide (Arg-Trp-(d-Arg)-Asn-Arg-His-His-His-His-His-His) and B3int (Arg-Trp-(d-Arg)-Asn-Arg) were obtained from Dangang Biological Co., Ltd. (Hangzhou, China). B16 cells and L929 cells were purchased from the Institute of Biochemistry and Cell Biology, Chinese Academy of Sciences (Shanghai, China). RPMI 1640 medium, DMEM high-glucose medium, phosphate-buffered saline (PBS), trypsin, and fetal bovine serum were supplied by Gibco Life Technologies (Grand Island, NY, USA). 3-(4,5-Dimethylthiazole-2)-2,5-diphenyltetrazolium bromide (MTT), 4′,6-diamino-2-phenylindole (DAPI), annexin V–FITC/PI cell apoptosis analysis kits, and lysosome staining kits (green fluorescence) were provided by KeyGEN Bio TECH Co., Ltd. (Nanjing, China). Calcein acetoxymethyl ester (calcein AM) and propidium iodide (PI) were purchased from Sigma-Aldrich (St. Louis, MO, USA). A reactive oxygen species assay kit (DCFH-DA) was obtained from Beyotime Biotechnology (Shanghai, China). TUNEL and H&E commercial detection kits were also obtained from Beyotime (Beijing, China).

### 2.1. Preparation of MSN and MSN−NH_2_

CTAB (2 g) and TEA (150 μL) were sequentially dispersed in H_2_O (20 mL) and stirred vigorously at 95 °C. After 1 h, TEOS (1.5 mL) was added to the solution dropwise, and the mixture was stirred for another 1 h. After recovery by centrifugation, the solid product was washed with ethanol several times and dried under vacuum at 60 °C. MSNs were obtained via calcination in a muffle furnace at 550 °C for 3 h.

The prepared MSNs (50 mg) were next dispersed in ethanol (50 mL). APTES (3 mL) was added, and the mixture was stirred at room temperature for 24 h. After recovery by centrifugation, the solid product was washed with ethanol several times and dried under vacuum to obtain MSN−NH_2_ NPs.

### 2.2. Preparation of MSN−HtB

HtB peptide (20 mg) was dissolved in PBS (pH = 7.4, 10 mL) containing EDC (60 mg) and NHS (40 mg), and stirred for 2 h to activate the peptide. Then, the MSN−NH_2_ NPs (25 mg) were dispersed in the HtB solution, and the mixture was stirred at room temperature for 24 h. The resultant suspension was dialyzed with deionized water for 48 h (MWCO: 3500 Da) and then lyophilized.

### 2.3. Preparation of CP/CQ@MSN−HtB/Cu^2+^

CQ (8 mg) and CP (8 mg) were dissolved in an aqueous saline solution (0.9%, *w*/*v*, 10 mL), and then added to a dispersion of MSN−HtB NPs (1 mg·mL^−1^) in PBS (10 mL). After stirring for 12 h at room temperature, CuCl_2_ (0.5 g) was added, and the reaction mixture was stirred for a further 24 h. After this, the solid product was dialyzed with deionized water for 48 h (MWCO: 3500 Da) and then lyophilized, resulting in CP/CQ@MSN−HtB/Cu^2+^ NPs. Control nanoparticles were prepared, in which drug-free nanoparticles were sealed with Cu^2+^; these were termed MSN−HtB/Cu^2+^ NPs, and were generated by directly chelating Cu^2+^ to the MSN−HtB NPs without first drug-loading them. CP@MSN-HtB/Cu^2+^ NPs were prepared as above but with the omission of CQ from the reaction mixture.

### 2.4. Materials’ Characterization

The morphologies of the nanoparticles were studied by transmission electron microscopy (TEM) on a JEM-2100UHR instrument (JEOL, Tokyo, Japan). Particle size and zeta potential data were obtained on a Zetasizer Nano ZS90 instrument (Malvern Instruments, Malvern, UK). Infrared absorption spectra were collected on a Nicolet Nexus 870 spectrometer (Thermo Fisher, Waltham, MA, USA). UV–Vis absorption spectra were collected from 200 to 1000 nm using a UV 3600 spectrophotometer (Shimadzu, Kyoto, Japan) at ambient temperature. Thermogravimetric analysis (TGA) was performed on a TGA-101 analyzer (JB, Shanghai, China). Pore size and specific surface area were quantified on a TriStar 3000 automatic nitrogen adsorption specific surface area analyzer (Micromeritics Instruments Corporation, Atlanta, GA, USA). X-ray photoelectron spectroscopy (XPS) was used to probe the surface chemical composition, with experiments carried out using an ESCALAB 250Xi X-ray photoelectron spectrometer (Thermo Fisher, Waltham, MA, USA). Elemental composition was determined on a 710-ES inductively coupled plasma–optical emission spectrometer (ICP–OES; Varian, Salt Lake City, UT, USA). For the latter, the samples were digested in HNO_3_ and then diluted into the calibration range with deionized water.

To determine the percentage encapsulation efficiency (EE) and drug loading (DL) of CP and CQ in CP/CQ@MSN−HtB at the end of the CP/CQ loading process, the NP dispersion in PBS was centrifuged to precipitate any solid material, and the amount of unencapsulated drug in the supernatant was determined by UV–Vis spectroscopy. The CP and CQ concentrations were quantified with reference to a predetermined calibration curve. In the case of CP, the supernatant was first mixed with an equal volume of o-phenylenediamine (OPD), and heated at 95 °C for 10 min prior to UV–Vis measurements [47]. The *EE* and *DL* were calculated as follows [48,49,50]:

   *EE* = (weight of drug loaded in MSNs/total weight of drug) × 100% *DL* = (weight of drug loaded in MSNs/total weight of NPs)

### 2.5. Chelation of Cu^2+^ with His-Tag

The chelation of Cu^2+^ with the His-tag was assayed using pyrocatechol violet (PV). HtB peptide (2 mg) and MSN−HtB NPs (5 mg) were dispersed in sodium acetate buffer solution (1 mL) at pH 6.0. The resultant mixture (200 μL) was combined with sodium acetate buffer solution (2.7 mL), PV solution (4 mmol·L^−1^, 60 μL, in pH 6 sodium acetate buffer), and CuSO_4_·5H_2_O solution (4 mmol·L^−1^, 100 μL, in pH 6 sodium acetate buffer), mixed well, and left to stand for 15 min. The chelation of Cu^2+^ and the His-tag results in insoluble agglomerates [51] were quantified by UV-Vis spectroscopy.

### 2.6. In Vitro Release Experiments

In vitro release of CP or CQ from the CP/CQ@MSN−HtB/Cu^2+^ NPs was studied by dispersing CP/CQ@MSN−HtB/Cu^2+^ NPs (5 mg) in 2 mL of two different buffer solutions (PBS at pH 7.4 or 5.0) placed in a dialysis bag (MWCO: 3500 Da). This was then dialyzed against PBS (20 mL) at the appropriate pH at 37 °C, under stirring. At specific time intervals, the dialysate (2 mL) was removed and replaced with the same amount of preheated PBS. The amount of released CQ was determined by UV–Vis spectroscopy at *λ* = 343 nm. CP quantification was performed by mixing the release aliquot with OPD (1.2 mg·mL^−1^, 2 mL) in a 9% *w*/*v* aqueous NaCl solution at 95 °C. This mixture was heated for 10 min to form a complex, and analyzed with a UV–Vis spectrophotometer at *λ* = 705 nm.

### 2.7. Evaluation of Extracellular ·OH Production Capacity

Methylene blue (MB) is a dye that can be degraded by ·OH, and can thus be used as an indicator for the generation of ·OH [52]. Experiments were set up at 37 °C under stirring, as follows: (1) PBS (3 mL, pH 5.0) with MB (10 μg·mL^−1^); (2) PBS (3 mL, pH 5.0) with MB (10 μg·mL^−1^) and H_2_O_2_ (8 mM); (3) PBS (3 mL, pH 5.0) with MB (10 μg·mL^−1^) and CP/CQ@MSN−HtB/Cu^2+^ NPs (0.3 mg·mL^−1^); (4) PBS (3 mL, pH 5.0) with MB (10 μg·mL^−1^), H_2_O_2_ (8 mM), and CP/CQ@MSN−HtB/Cu^2+^ NPs (0.3 mg·mL^−1^); (5) PBS (3 mL, pH 7.4) with MB (10 μg·mL^−1^), H_2_O_2_ (8 mM), and CP/CQ@MSN−HtB/Cu^2+^ NPs (0.3 mg·mL^−1^). The suspensions were stirred for 24 h, after which the absorbance at *λ* = 665 nm was measured.

### 2.8. Cell Culture

B16 cells were cultured in RPMI 1640 medium containing 10% (*v*/*v*) FBS and 1% (*v*/*v*) penicillin–streptomycin solution. L-929 cells were cultured in DMEM medium supplemented with 10% (*v*/*v*) FBS and 1% (*v*/*v*) penicillin–streptomycin solution. All of the cells were cultured in an atmosphere of 5% CO_2_ at 37 °C. Negative controls comprised cells treated with an equal volume of PBS (pH 7.4). Three independent experiments were performed, with five replicate wells per experiment.

### 2.9. Cytotoxicity Studies

The cytotoxicity of the nanoparticles to tumor cells was analyzed by the MTT assay. B16 cells were seeded in 96-well plates (1 × 10^5^ cells·mL^−1^, 100 μL) and incubated for 24 h at 37 °C before the media was aspirated. Subsequently, the cells were incubated with different concentrations of CP, CQ, and CP/CQ@MSN−HtB/Cu^2+^ NPs, and cultured in complete 1640 medium for another 12 h. Next, the MTT reagent (5 mg·mL^−1^, 100 μL) was added to each well, and the plate was incubated for 4 h. The media was aspirated, and DMSO (150 μL) added to each well of the plate, which was then incubated for another 20 min to dissolve the formazan crystals. Finally, the absorbance at 570 nm was measured by a microplate reader (Thermo Scientific Multiskan FC, Waltham, MA, USA).

The cytotoxicity of the nanoparticles to L-929 cells was also tested by MTT assay. The experimental method was the same as above, except that the cells were treated with different concentrations of MSN−HtB NPs and CP/CQ@MSN−HtB/Cu^2+^ NPs.

Calcein AM/PI double-labeling was employed to visualize the effects of the NPs on cells. B16 cells were seeded in 6-well plates (1 × 10^5^ cells·mL^−1^, 2 mL) and incubated for 24 h at 37 °C. The medium was then removed and replaced with fresh medium containing the test formulations. The cells were divided into 6 groups—(1) control; (2) free CP; (3) free CQ; (4) MSN−HtB NPs; (5) CP@MSN−HtB/Cu^2+^ NPs; and (6) CP/CQ@MSN−HtB/Cu^2+^ NPs—and incubated at an equivalent concentration of Cu^2+^ (10 μg·mL^−1^) or CP (30 μg·mL^−1^) for another 12 h. The cells were then washed with PBS three times and cultured in RPMI for a further 24 h. The cells were subsequently stained with a mixture of calcein-AM/PI solution for 10 min. Living cells (green) and apoptotic cells (red) were observed with an inverted fluorescent microscope (Nikon TE-2000U, Tokyo, Japan).

The annexin V–FITC and PI double-staining method was used to quantify cell death. B16 cells were seeded in 6-well plates (1 × 10^5^ cells·mL^−1^, 2 mL) and incubated for 24 h at 37 °C. The medium was removed and replaced with fresh medium containing the test formulations. The cells were divided into 6 groups—(1) control; (2) free CP; (3) free CQ; (4) MSN−HtB NPs; (5) CP@MSN−HtB/Cu^2+^ NPs; and (6) CP/CQ@MSN−HtB/Cu^2+^ NPs—and incubated for 12 h. Next, the cells were trypsinized (0.25% trypsin without EDTA), collected, and rinsed three times with PBS (pH 7.4). They were re-suspended with binding buffer (500 μL) at a concentration of 1 × 10^6^ cells·mL^−1^. An annexin V–FITC/PI cell apoptosis analysis kit was used to stain the cells according to the manufacturer’s protocol, and the samples were quantified by flow cytometry (BD FACS Calibur, Franklin Lakes, NJ, USA).

### 2.10. Cellular Uptake

In order to study the cellular uptake of NPs, fluorescent DOX was loaded in the MSNs instead of CP and CQ, following the same protocol as detailed above. To check the influence of the His-tag on uptake, control nanoparticles in which B3int was conjugated to the NP surface were also prepared. These were produced by replacing HtB with B3int in the MSN synthesis procedure, and then loading with DOX. The cellular uptake of DOX@MSN−HtB/Cu^2+^ NPs was evaluated by confocal laser scanning microscopy (CLSM, Nikon C1-si, Tokyo, Japan). B16 and L-929 cells were separately plated on glass-bottomed culture dishes (1 × 10^5^ cells·mL^−1^, 2 mL) for 24 h at 37 °C. The media was aspirated, and then replaced with fresh media containing the test formulations. The cells were divided into 4 groups—(1) control; (2) free DOX (0.5 μg·mL^−1^); (3) DOX@MSN−B3int NPs (DOX: 0.5 μg·mL^−1^); and (4) DOX@MSN−HtB/Cu^2+^ NPs (DOX: 0.5 μg·mL^−1^)—and incubated in complete medium for another 4 h. After removal of the medium and washing with PBS, the cells were stained with a lysosome staining kit for 45 min, fixed with aqueous formaldehyde (4%, *v*/*v*) at room temperature, stained with DAPI for 3 min, and then imaged by CLSM.

### 2.11. Intracellular ROS Generation

Intracellular total ROS generation in B16 cells was detected using a DCFH-DA ROS assay kit. Cells were seeded in 6-well plates (1 × 10^5^ cells·mL^−1^, 2 mL) and incubated for 24 h at 37 °C. After removing the medium and replenishing it with fresh medium containing the test formulations, five groups of experiments were performed as follows: (1) control; (2) CuCl_2_; (3) MSN−HtB-Cu^2+^ NPs; (4) CP/CQ@MSN−HtB NPs; and (5) CP/CQ@MSN−HtB/Cu^2+^ NPs. The formulations were added to give equivalent concentrations of Cu^2+^ (10 μg·mL^−1^) or CP (30 μg·mL^−1^), and the cells were incubated for another 12 h. Next, the culture medium was replaced with fresh RPMI containing DCFH-DA (10 μM) for another 30 min of incubation. Afterwards, the cells were washed three times with PBS. Finally, the intensity of green fluorescence was observed using an inverted fluorescence microscope.

### 2.12. In Vivo Biodistribution

All animal experiments were undertaken following review and approval by the Experimental Animal Committee of Donghua University (SYXK(SH)2020-0018). C57BL/6 black mice (specific pathogen-free grade, 18–20 g) were acquired from Shanghai Jiesijie Experimental Animal Co., Ltd. (Shanghai, China). Tumors were implanted by subcutaneous injection of 2 × 10^6^ B16 cells in 100 µL of PBS (pH 7.2–7.4) into the right flank area of each mouse. The mice were randomized into two groups (5 mice each) when the tumor volume (determined as length × width^2^/2) reached ca. 100 mm^3^.

ICP–OES was used to determine the content of Pt and Cu, and to investigate in vivo biodistribution. CP/CQ@MSN−HtB/Cu^2+^ NPs or free CP in PBS solution (CP concentration: 1 mg·mL^−1^, 200 μL) were injected into the tail vein. The mice were euthanatized after 24 h, and the tumors and major organs (i.e., heart, liver, spleen, lungs, and kidneys) were collected and weighed before being digested with aqua regia. After diluting the digestion solution 10 times with water, the contents of Pt and Cu were determined by ICP–OES.

### 2.13. In Vivo Antitumor Efficacy and Biosafety

Tumor-bearing mice with tumor volumes of ca. 100 mm^3^ were randomly divided into six groups (*n* = 5), and injected intravenously through the tail vein with the formulations under test (200 μL). The following treatments were investigated: (1) PBS, (2) free CP, (3) free CQ, (4) MSN−HtB/Cu^2+^ NPs, (5) CP/CQ@MSN−HtB NPs, and (6) CP/CQ@MSN−HtB/Cu^2+^ NPs. The amount of CP in each treatment was set at 300 μg·kg^−1^. During the experiment, the tumor volume and body weight were measured every three days.

### 2.14. Histological Analysis

After 12 days, mice were euthanatized by cervical dislocation. The tumor tissues and major organs (i.e., the heart, lungs, spleen, liver, and kidneys) were excised from the mice and fixed in 4% aqueous formaldehyde. They were then embedded in paraffin and sliced into 5 μm sections. These sections were stained with hematoxylin and eosin (H&E) and studied under an optical microscope. In addition, TUNEL (TdT-mediated dUTP-biotin nick end labeling) staining was used to analyze apoptosis.

### 2.15. Kaplan–Meier Analysis

Additional tumor-bearing mice with tumor volumes of 100 mm^3^ were employed for Kaplan–Meier analysis. These mice were divided into the abovementioned 6 groups (*n* = 10), and injected intravenously through the tail vein with the formulations under test (200 μL). The survival status was recorded every day for 20 days.

### 2.16. Statistical Analysis

Statistical analyses were performed using either Student’s *t*-test or one-way ANOVA analysis, followed by a Newman–Keuls test; *p*-values < 0.05 were set as statistically significant findings, and the data were marked with (*) for *p* < 0.05, (**) for *p* < 0.01, and (***) for *p* < 0.001.

## 3. Results and Discussion

### 3.1. Synthesis and Characterization of CP/CQ@MSN−HtB/Cu^2+^ NPs

**Figure 1:**
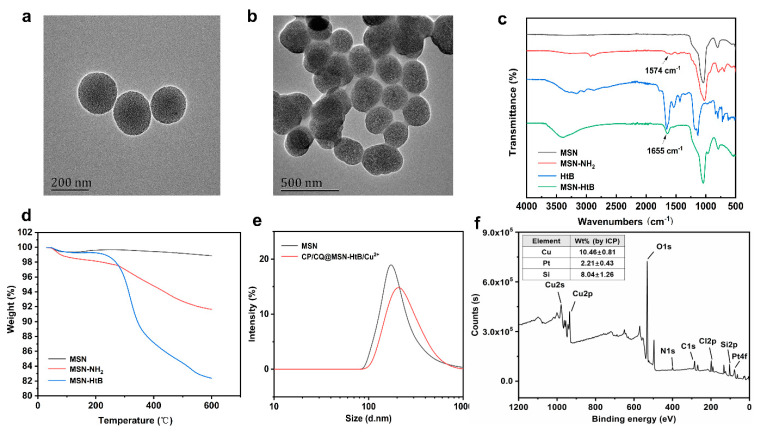
(**a**) TEM images of MSNs and (**b**) CP/CQ@MSN−HtB/Cu^2+^. (**c**) IR spectra of functionalized MSNs and the HtB peptide. (**d**) TGA curves. (**e**) Particle sizes of MSN and CP/CQ@MSN−HtB/Cu^2+^ NPs. (**f**) Survey XPS spectrum and ICP data of CP/CQ@MSN−HtB/Cu^2+^.

The MSN morphology was first investigated by TEM (Figure 1a). The nanoparticles were spherical, and about 160 nm in size. The mesopores were dense and distributed evenly throughout the nanoparticles. N_2_ adsorption–desorption isotherms confirmed the mesoporous structure of the MSNs, and showed that the pore size was ca. 2.6 nm (Supplementary Materials, Figure S1).

Next, the surface of the nanoparticles was modified. The MSNs were first functionalized via co-condensation to obtain MSN−NH_2_ [53]. Amidation between MSN−NH_2_ and the carboxyl group in HtB peptides then allowed the generation of MSN−HtB. The FTIR spectra of MSN−NH_2_ and MSN−HtB (Figure 1c) showed the bending vibration peak of N–H bonds at 1574 cm^−1^ and the stretching vibration peak of C=O bonds at 1655 cm^−1^, respectively, confirming the successful incorporation of amino groups and HtB peptides onto the surface. Changes in zeta potential also validated the functionalization (Figure S2). The zeta potential of the initial MSNs was −28.4 ± 1.5 mV, but this increased to +39.4 ± 1.0 mV after modification with APTES, and changed again to 24.1 ± 1.1 mV after connecting the HtB peptide.

To determine the HtB peptide content on the MSN surface, thermogravimetric analysis (TGA) was performed to analyze the weight loss (Figure 1d). The decrease in mass below 100 °C arose from the evaporation of H_2_O. The mass of MSN−NH_2_ and MSN−HtB was reduced by 7.2% and 16.5%, respectively, compared with MSN between 100 °C and 600 °C, indicating that the content of grafted HtB peptide was 9.3% *w*/*w*.

CP and CQ were next loaded into the MSN−HtB NPs, and Cu^2+^ was chelated onto the surface to seal the pores. UV spectra are shown in Figure S3. Comparing the spectra of CP, CQ, and CP/CQ@MSN−HtB, we can see that MSN−HtB was successfully loaded with CQ, as the characteristic peaks of CQ (e.g., at 343 nm) can be seen. The CP peaks are hard to distinguish from those of CQ. After Cu capping, the CQ peaks are less distinct, likely owing to absorption by the Cu–HtB complex. This indicates that the drugs were successfully sealed in the pores. After drug loading and sealing, the pores of the NPs remained intact (Figure 1b), and the particle size increased from 160 to 200 nm (Figure 1e). Figure 1f depicts XPS data. The peaks of Cu, Pt, and Cl are all visible in the XPS spectrum, confirming the presence of these elements in the NPs. The presence of Pt in the formulation confirms CP loading. ICP analysis revealed that the content of Cu was 10.5 ± 0.8 mg·g^−1^ and the content of Pt was 2.2 ± 0.4 mg·g^−1^ (Figure 1f). UV analysis shows that the DL of CP is 381 ± 13 μg·mg^−1^ and the EE is 84.1 ± 5.8 %. The DL of CQ is 178.5 ± 9.4 μg·mg^−1^, and the EE is 75.9 ± 7.2 %.

The chelation of Cu^2+^ with the His-tag was probed using a PV assay. After adding Cu^2+^ to HtB peptides and MSN−HtB NPs, insoluble flocculent precipitates were generated (Figure S4a). In the corresponding UV spectra, the redshift of the phenolic absorbance peak in the mixture from 430 nm to 655 nm indicates the production of oligomers (Figure S4b). This clearly evidences successful chelation of Cu^2+^ by the HtB on the NPs.

### 3.2. In Vitro Drug Release

**Figure 2:**
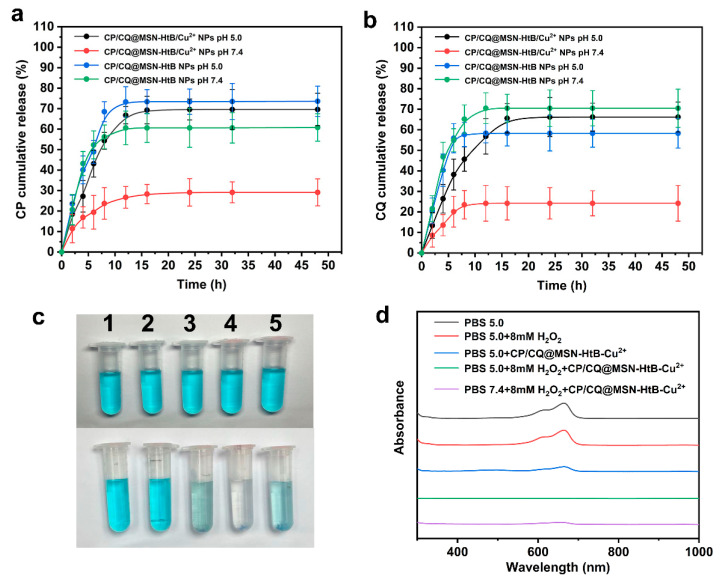
In vitro (**a**) CP and (**b**) CQ release profiles from CP/CQ@MSN−HtB and CP/CQ@MSN−HtB/Cu^2+^ at different pH values. (**c**) Images of MB solutions with different additives: (1) PBS, pH 5.0; (2); PBS, pH 5.0, + 8 mM H_2_O_2_; (3) PBS, pH 5.0 + CP/CQ@MSN−HtB/Cu^2+^ NPs; (4) PBS, pH 5.0 + 8 mM H_2_O_2_ + CP/CQ@MSN−HtB/Cu^2+^ NPs; (5) PBS, pH 7.4 + 8 mM H_2_O_2_, + CP/CQ@MSN−HtB/Cu^2+^ NPs—top: freshly prepared; bottom: after aging; and (**d**) the corresponding UV–Vis absorption spectra.

The complex of His-tag and Cu^2+^ chelates present in alkaline conditions (pH = 7–8) collapses in acidic environments (pH = 4–6) [54]. Drug release data are presented in Figure 2a,b. Approximately 20–30% of the CP and CQ loading was released at pH 7.4. CP and CQ were released to a greater extent at pH 5.0, indicating that pore sealing by Cu^2+^ is reversed in an acidic environment. Thus, the formulations have pH-responsive drug release, and are likely to free more of their cargo in the TME than under normal physiological conditions.

### 3.3. Evaluation of Extracellular ·OH Production

Methylene blue (MB) was used as an indicator to explore the effect of CP/CQ@MSN−HtB/Cu^2+^ on the production of ·OH in vitro. ·OH has the ability to destroy the conjugated structure of organic dyes and, thus, cause color fading. Therefore, the production of ·OH can be detected by measuring the change in the absorbance of MB [55,56]. After adding H_2_O_2_ for 24 h, the MB solution did not fade, indicating that MB was stable at this concentration of H_2_O_2_ [57]. As demonstrated in Figure 2c, at pH = 5, the color of an MB solution containing H_2_O_2_ almost completely faded after adding CP/CQ@MSN−HtB/Cu^2+^ NPs. This was because Cu^2+^ released in the acidic environment can react with H_2_O_2_ to produce ·OH. In contrast, at pH 7.4, the MB solution faded to a notably lesser degree. This may be due to much of the Cu^2+^ remaining chelated, with a reduced amount being released into solution. The UV spectra confirm the visual observations (Figure 2d). The MB absorption peak at 665 nm disappeared at pH 5.0 in the presence of the NPs and H_2_O_2_, indicating that in an acidic environment the nanoparticles can effectively release Cu^2+^. This effect was reduced at pH 7.4.

### 3.4. Intracellular Uptake

**Figure 3:**
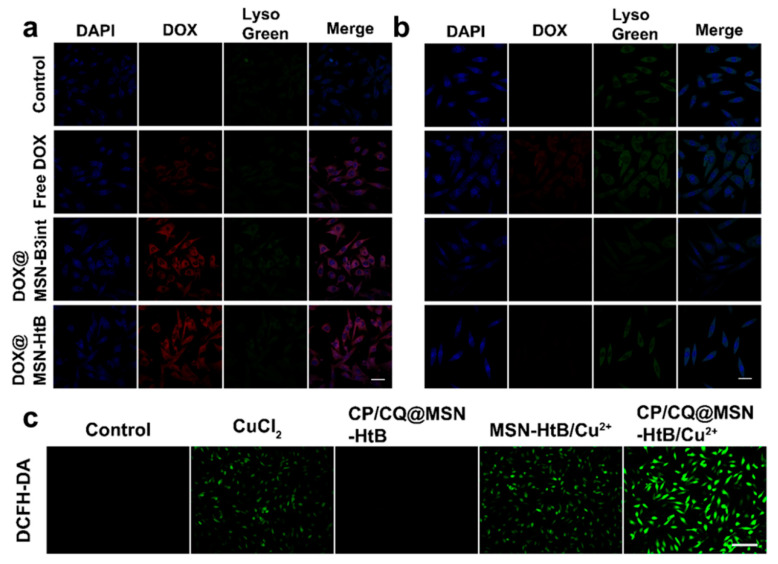
CLSM images of (**a**) B16 and (**b**) L-929 cells treated with DOX, DOX@MSN−B3int, and DOX@MSN−HtB. Scale bars: 25 μm. (**c**) Fluorescence images of B16 cells co-stained with DCFH-DA after different treatments. Scale bar: 25 μm.

CP and CQ cannot easily be seen by fluorescent microscopy. Hence, the nanoparticles were loaded with the fluorescent drug DOX to detect cellular uptake. The ability of DOX@MSN−HtB NPs to target cancer cells was evaluated in B16 and L-929 cells by CLSM. The resultant images (Figure 3a,b) revealed that both cell lines showed a certain amount of free DOX uptake (as evidenced by red intracellular fluorescence). In addition, it was observed that the intracellular DOX fluorescence of cells exposed to DOX@MSN−HtB NPs was stronger than that of those receiving free DOX, and was similar to that of cells given DOX@MSN−B3int NPs. The results show that DOX@MSN−HtB NPs can target B16 cells, and that the targeting ability of the B3int peptide is not affected by His-tag. The red DOX overlaps with the green fluorescence of the lysosome, suggesting that the NPs could be located within the lysosomes. In contrast, there was almost no fluorescence with L-929 cells treated with DOX@MSN−B3int and DOX@MSN−HtB (Figure 3b). This is because there is no overexpressed integrin β3 on the surface of L-929 cells and, thus, no active targeting.

### 3.5. Intracellular ·OH Detection

Since the DCFH-DA probe can be oxidized to DCF—a green fluorescent molecule—under the action of ROS, it can be used to detect ROS produced in the cell. Figure 3c reveals that in the control group and CP/CQ@MSN−HtB group there was almost no fluorescence in the cells, due to the absence of Cu^2+^. Cells treated with CuCl_2_ or MSN−HtB/Cu^2+^ produced relatively weak fluorescence, due to some ROS being generated by the Cu^2+^ present. The CP/CQ@MSN−HtB/Cu^2+^ group gave markedly enhanced fluorescence, indicating that the NPs successfully released CP and Cu^2+^ in the acidic environment of the lysosome. The presence of CP and Cu^2+^ aids Fenton-reaction-mediated production of ROS [58].

### 3.6. Cytocompatibility and Cytotoxicity

**Figure 4:**
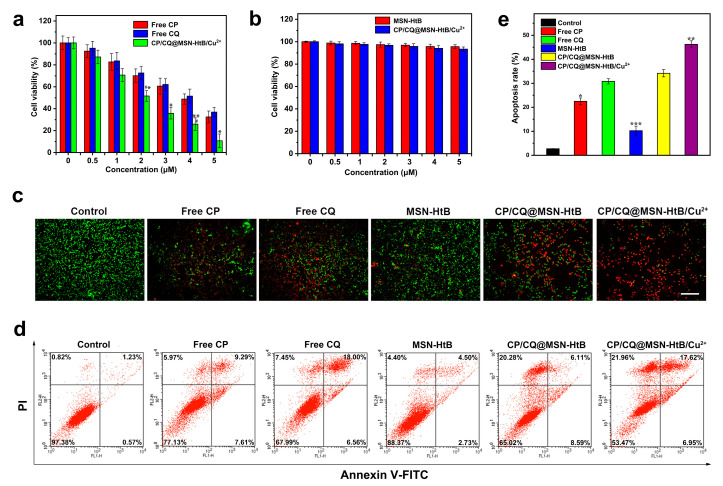
Cell viability of (**a**) B16 cells and (**b**) L-929 cells after incubation with MSN−HtB and CP/CQ@MSN−HtB/Cu^2+^ NPs for 24 h. (**c**) Fluorescence images of B16 cells co-stained with calcein AM/PI after different treatments. Scale bar: 100 μm. (**d**) Flow cytometry analysis using annexin V–FITC/PI staining, and (**e**) statistical analysis (results are presented as mean ± SD, *n* = 3). (* *p* < 0.05, ** *p* < 0.01, *** *p* < 0.001, compared with free CQ; # *p* < 0.05, compared with free CP).

B16 cells were used to evaluate the cytotoxicity of the CP/CQ@MSN−HtB/Cu^2+^ NPs (Figure 4a). Compared with free CP, CP/CQ@MSN−HtB/Cu^2+^ NPs showed greater toxicity to B16 cells after 24 h of incubation. Similarly, the cytotoxicity of CP/CQ@MSN−HtB/Cu^2+^ NPs was also higher than that of free CQ. These results can be explained by dint of the CP/CQ@MSN−HtB/Cu^2+^ NPs releasing their drug cargo and providing synergistic therapy. Healthy L-929 cells were used to assess the biocompatibility of the nanoparticles. As shown in Figure 4b, CP/CQ@MSN−HtB/Cu^2+^ NPs induced negligible cytotoxicity to L-929 cells even when the material concentration was as high as 5 μg·mL^−1^, indicating that the CP/CQ@MSN−HtB/Cu^2+^ NPs have good biocompatibility.

Calcein AM/PI double-staining was used to evaluate the apoptosis of B16 cells (Figure 4c). Compared with free CP and CQ, the cells treated with CP/CQ@MSN−HtB/Cu^2+^ NPs showed strong red fluorescence (apoptotic cells), confirming that the NPs can effectively transport CP and CQ to the cells and cause cell apoptosis. The cells treated with CP/CQ@MSN−HtB NPs showed stronger green fluorescence (living cells) than those exposed to CP/CQ@MSN−HtB/Cu^2+^ NPs, indicating that Cu^2+^ can augment the chemotherapy provided by CP and CQ, proving synergistic chemotherapy/chemodynamic therapy. In addition, the MSN−HtB group had negligible red fluorescence, indicating that MSN−HtB has almost no biological toxicity.

Flow cytometry analysis was used to confirm the results of these assays (Figure 4d,e). In the PBS-treated group, 97.4% of the cells survived. By contrast, the apoptotic rates of the free CP and CQ groups were 22.9% and 32.0%, respectively, while the equivalent value for the CP/CQ@MSN−HtB group was 35.0%; this indicates that CP and CQ can effectively be transported into the cell via the NP carrier and cause apoptosis. In the CP/CQ@MSN−HtB/Cu^2+^ group, 46.5% of the cells were apoptotic. This increased percentage is attributable to the CDT effects induced by Cu^2+^. The cell survival rate after treatment with MSN−HtB was 88.4%, consistent with the results above, and showing the NPs also have only slight cytotoxicity.

### 3.7. In Vivo Biodistribution

**Figure 5:**
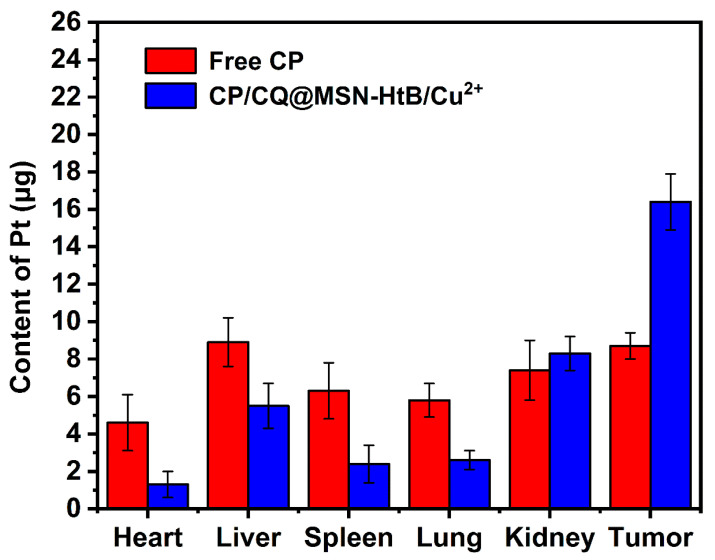
Contents of Pt in the major organs and tumor cells after intravenous injection of CP and CP/CQ@MSN−HtB/Cu^2+^ NPs.

Free CP or CP/CQ@MSN−HtB/Cu^2+^ NPs were injected into mice through the tail vein. After 24 h, the contents of Pt and Cu in the heart, liver, spleen, lungs, kidneys, and tumor cells were measured by ICP–OES. Both Pt and Cu could be seen to be concentrated in the tumor (Figure 5 and Figure S5). Compared with an injection of an equivalent amount of free CP, the content of Pt in the tumor site increased markedly when using CP/CQ@MSN−HtB/Cu^2+^ NPs (Figure 5), indicating that the nanoparticles were enriched in the tumor site more effectively than the free drug. This can be attributed to the targeting ability of the NPs. The levels of Cu and Pt in the liver and kidneys were also relatively high. The former was due to the uptake of CP/CQ@MSN−HtB/Cu^2+^ NPs by the mononuclear phagocyte system, while the latter resulted from renal excretion [59].

### 3.8. In Vivo Antitumor Efficacy and Biosafety

**Figure 6:**
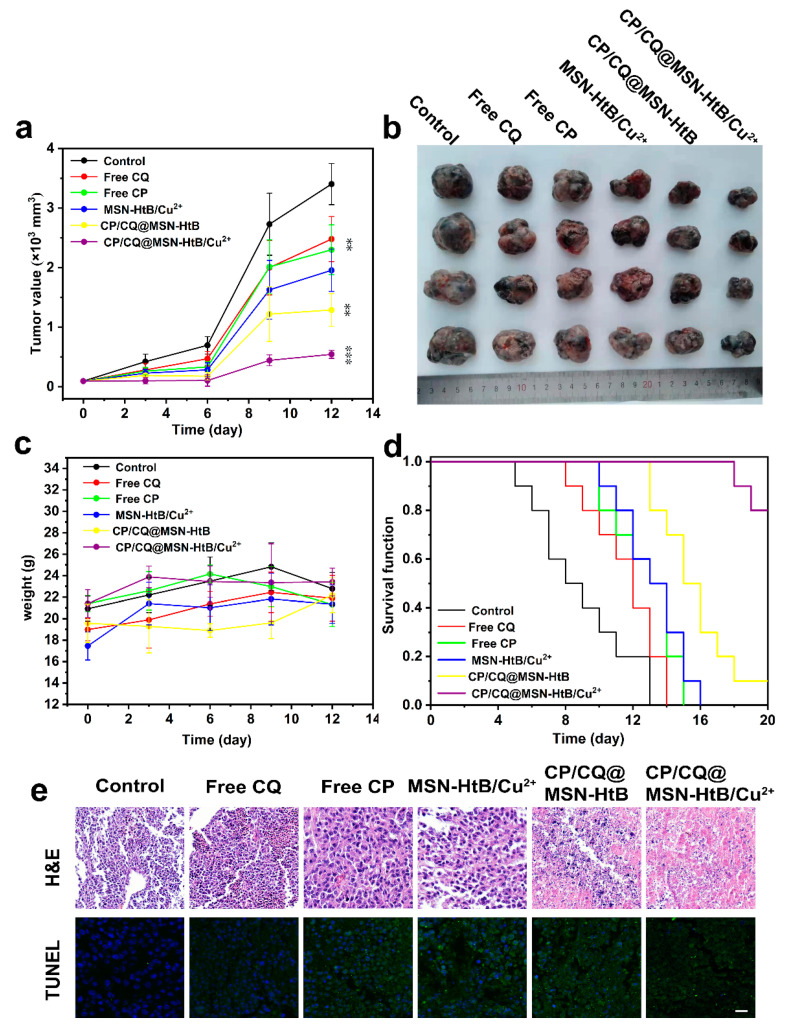
The results of in vivo antitumor studies: (**a**) The tumor volume growth curves (** *p* < 0.01, *** *p* < 0.001) and (**b**) representative photographs of tumors isolated on day 12. (**c**) Average body weight changes. (**d**) Kaplan–Meier survival curves. (**e**) H&E staining and TUNEL staining of tumor sections. Scale bars: 20 μm.

We investigated the therapeutic performance of CP/CQ@MSN−HtB/Cu^2+^ NPs using B16 melanoma tumor-bearing mice. After the tumors had grown to ca. 100 mm^3^ in volume, mice were injected with PBS, free CQ, free CP, MSN−HtB/Cu^2+^, CP/CQ@MSN−HtB, and CP/CQ@MSN−HtB/Cu^2+^ NPs. Tumor volume data (Figure 6a) show that the tumor growth of mice treated with CP/CQ@MSN−HtB/Cu^2+^ was significantly inhibited. The CP/CQ@MSN−HtB treatment group also displayed a certain degree of tumor growth inhibition but, due to the lack of Cu^2+^-mediated CDT, this was less marked than with CP/CQ@MSN−HtB/Cu^2+^.

The mice were euthanatized and the tumors were excised at day 12 (Figure 6b). The photographs revealed that the tumor shrank much more after treatment with CP/CQ@MSN−HtB/Cu^2+^ compared with the other groups. When these tumors were weighed (Figure S6), mice treated with CP/CQ@MSN−HtB/Cu^2+^ had an average tumor mass of ca. 2.74 g—notably less than with the free CP (5.79 g) or free CQ treatment groups (6.03 g).

Body weight data are given in Figure 6c. There was no significant weight loss in any of the treatment groups, indicating that the CP/CQ@MSN−HtB/Cu^2+^ NPs have good biocompatibility and are well tolerated, with minimal off-target side effects. In addition, the survival time of tumor-bearing mice was evaluated using the Kaplan–Meier test (Figure 6d). The survival curves show that 80% of the mice treated with nanoparticles survived the 20-day experimental period. All of the mice treated with PBS, free CP, and free CQ died within 15 days. The survival time of mice treated with CP/CQ@MSN−HtB/Cu^2+^ nanoparticles was notably higher than that of other groups.

### 3.9. Histological Analysis

The tumors were processed for histological analysis, including H&E and TUNEL staining (Figure 6e). In H&E staining, the tumor tissue of the CP/CQ@MSN−HtB/Cu^2+^ treatment group showed a large area of tissue necrosis, indicated by pink/red-stained unstructured areas, and the cell nuclei (blue) were barely visible. There was no significant tissue necrosis in the free CP and free CQ treatment groups, which was attributed to their being distributed throughout the body rather than being localized in the tumor. The necrotic area of the tumor tissue increased greatly after the addition of Cu^2+^. TUNEL staining (Figure 6e) revealed that the number of apoptotic cells (green) in the tumor tissue after the CP/CQ@MSN−HtB/Cu^2+^ treatment was greater than that in the CP/CQ@MSN−HtB treatment group, while the TUNEL positive signal in the other groups was markedly lower still. This confirms the potent synergistic chemotherapy/CDT properties of CP/CQ@MSN−HtB/Cu^2+^.

Tissue sections of the heart, liver, spleen, lungs, and kidneys were additionally subjected to H&E staining (Figure S7). The results showed no obvious pathological abnormalities, indicating that the NP formulations had good selectivity and histocompatibility.

In this work, we designed and prepared a Cu^2+^-chelating mesoporous silica drug delivery system and evaluated its antitumor efficacy. Compared with other mesoporous silicon-based drug delivery systems, this has a higher cisplatin drug-loading and entrapment efficiency [60,61]. Cell experiments show that the formulation has good biocompatibility. In contrast, previously reported peptide-sealed mesoporous silica NPs showed a degree of cytotoxicity [21]. The results of in vivo experiments confirm that the CP/CQ@MSN−HtB/Cu^2+^ NPs provide effective cisplatin/Cu^2+^ synergistic therapy against cancer, which is consistent with other results in the literature [62].

## 4. Conclusions

In this work, we designed pH-responsive drug-loaded nanoparticles allowing synergistic chemotherapy/chemodynamic therapy of tumors. These were based on mesoporous silica nanoparticles (NPs) loaded with cisplatin and chloroquine. The NPs were functionalized with the targeting ligand His-tag-B3int, and the NP pores were sealed via the chelation of Cu^2+^ with the His-tag. Drug release was found to be accelerated in slightly acidic pH, where the Cu^2+^ was freed into solution and the NP pores opened. The NPs were shown to be able to generate ROS, and to be selectively taken up by cancer cells in vitro, where they appeared to be localized in the lysosomes. The presence of chloroquine then caused lysosomal membrane permeability, and released cisplatin and Cu^2+^ into the cytoplasm for synergistic chemotherapy/chemodynamic therapy. In vivo results showed the therapeutic potential of this approach. The NP platform developed is selectively trafficked to the tumor, where it leads to marked increases in cell apoptosis and a reduction in tumor volume over time. No evident off-target toxicity was noted. Thus, this work offers a new strategy to develop smart drug delivery systems using mesoporous silica nanoparticles as nanocarriers for multidimensional cancer therapy.

## Data Availability

Raw data are available from the corresponding authors opon request.

## Supplementary Materials

The following supporting information can be downloaded at: https://www.mdpi.com/article/10.3390/pharmaceutics14061200/s1, Figure S1: (a) N_2_ absorption-desorption isotherms and (b) corresponding pore-size distribution of the MSNs; Figure S2: Zeta potential results of MSN, MSN−NH_2_ and MSN−HtB; Figure S3: UV-Vis absorption spectra of free CP, CQ, MSN−HtB, CP/CQ@MSN−HtB and CP/CQ@MSN−HtB/Cu^2+^; Figure S4: (a) Visual detection of chelation between Cu^2+^ and the His-tag by PV (top: immediately after the addition of PV; bottom: after standing for 15 min) and (b) the corresponding UV/vis spectra; Figure S5: Content of Cu in the major organs and tumor after intravenous injection of CP/CQ@MSN−HtB/Cu^2+^ NPs; Figure S6: The tumor weights after the 12 day in vivo experimental period; Figure S7: H&E staining of major organs from the control and experimental groups.

## References

[1] Zhu C.L., Lu C.H., Song X.Y., Yang H.H., Wang X.R. (2011). Bioresponsive Controlled Release Using Mesoporous Silica Nanoparticles Capped with Aptamer-Based Molecular Gate. J. Am. Chem. Soc..

[2] Suteewong T., Sai H., Cohen R., Wang S., Bradbury M., Baird B., Gruner S.M., Wiesner U. (2011). Highly Aminated Mesoporous Silica Nanoparticles with Cubic Pore Structure. J. Am. Chem. Soc..

[3] Lin Y.S., Haynes C.L. (2009). Synthesis and Characterization of Biocompatible and Size-Tunable Multifunctional Porous Silica Nanoparticles. Chem. Mater..

[4] Corbalan J.J., Medina C., Jacoby A., Malinski T., Radomski M.W. (2012). Amorphous Silica Nanoparticles Aggregate Human Platelets: Potential Implications for Vascular Homeostasis. Int. J. Nanomed..

[5] Mal N.K., Fujiwara M., Tanaka Y. (2003). Photocontrolled Reversible Release of Guest Molecules from Coumarin-Modified Mesoporous Silica. Nature.

[6] Cai Y., Deng T., Pan Y., Zink J.I. (2020). Use of Ferritin Capped Mesoporous Silica Nanoparticles for Redox and pH Triggered Drug Release In Vitro and In Vivo. Adv. Funct. Mater..

[7] Jia L., Li Q., Zhang J., Huang L., Qi C., Xu L., Liu X., Wang G., Wang L., Wang Z. (2017). Safe and Effective Reversal of Cancer Multidrug Resistance Using Sericin-Coated Mesoporous Silica Nanoparticles for Lysosome-Targeting Delivery in Mice. Small.

[8] Xiao D., Hu J.J., Zhu J.Y., Wang S.B., Zhuo R.X., Zhang X.Z. (2016). A Redox-Responsive Mesoporous Silica Nanoparticle with a Therapeutic Peptide Shell for Tumor Targeting Synergistic Therapy. Nanoscale.

[9] Chen F., Hong H., Zhang Y., Valdovinos H.F., Shi S., Kwon G.S., Theuer C.P., Barnhart T.E., Cai W. (2013). In Vivo Tumor Targeting and Image-Guided Drug Delivery with Antibody-Conjugated, Radiolabeled Mesoporous Silica Nanoparticles. ACS Nano.

[10] Chen C., Ma T., Tang W., Wang X., Wang Y., Zhuang J., Zhu Y., Wang P. (2020). Reversibly-Regulated Drug Release Using Poly (tannic acid) Fabricated Nanocarriers for Reduced Secondary Side Effects in Tumor Therapy. Nanoscale Horiz..

[11] Wu J., Williams G.R., Niu S., Gao F., Tang R., Zhu L.M. (2019). A Multifunctional Biodegradable Nanocomposite for Cancer Theranostics. Adv. Sci..

[12] Li H., Tan L.L., Jia P., Li Q.L., Sun Y.L., Zhang J., Ning Y.Q., Yu J., Yang Y.W. (2014). Near-Infrared Light-Responsive Supramolecular Nanovalve Based on Mesoporous Silica-Coated Gold Nanorods. Chem. Sci..

[13] Lai C.Y., Trewyn B.G., Jeftinija D.M., Jeftinija K., Xu S., Jeftinija S., Lin V.S.Y. (2003). A Mesoporous Silica Nanosphere-Based Carrier System with Chemically Removable CdS Nanoparticle Caps for Stimuli-Responsive Controlled Release of Neurotransmitters and Drug Molecules. J. Am. Chem. Soc..

[14] Chen T., Wu W., Xiao H., Chen Y., Chen M., Li J. (2016). Intelligent Drug Delivery System Based on Mesoporous Silica Nanoparticles Coated with an Ultra-pH-Sensitive Gatekeeper and Poly (ethylene glycol). ACS Macro Lett..

[15] Lin J.T., Liu Z.K., Zhu Q.L., Rong X.H., Liang C.L., Wang J., Ma D., Sun J., Wang G.H. (2017). Redox-Responsive Nanocarriers for Drug and Gene co-Delivery Based on Chitosan Derivatives Modified Mesoporous Silica Nanoparticles. Colloids Surf. B.

[16] Patel K., Angelos S., Dichtel W.R., Coskun A., Yang Y.W., Zink J.I., Stoddart J.F. (2008). Enzyme-Responsive Snap-Top Covered Silica Nanocontainers. J. Am. Chem. Soc..

[17] Lei Q., Qiu W.X., Hu J.J., Cao P.X., Zhu C.H., Cheng H., Zhang X.Z. (2016). Multifunctional Mesoporous Silica Nanoparticles with Thermal-Responsive Gatekeeper for NIR Light-Triggered Chemo/Photothermal-Therapy. Small.

[18] Cho I.H., Shim M.K., Jung B., Jang E.H., Park M.J., Kang H.C., Kim J.H. (2017). Heat Shock Responsive Drug Delivery System Based on Mesoporous Silica Nanoparticles Coated with Temperature Sensitive Gatekeeper. Micropor. Mesopor. Mater..

[19] Chen W., Cheng C.A., Zink J.I. (2019). Spatial, Temporal, and Dose Control of Drug Delivery Using Noninvasive Magnetic Stimulation. ACS Nano.

[20] Wu J., Bremner D.H., Niu S., Wu H., Wu J., Wang H., Li H., Zhu L.M. (2018). Functionalized MoS_2_ Nanosheet-Capped Periodic Mesoporous Organosilicas as a Multifunctional Platform for Synergistic Targeted Chemo-Photothermal Therapy. Chem. Eng. J..

[21] Cheng Y.J., Zhang A.Q., Hu J.J., He F., Zeng X., Zhang X.Z. (2017). Multifunctional Peptide-Amphiphile End-Capped Mesoporous Silica Nanoparticles for Tumor Targeting Drug Delivery. ACS Appl. Mater. Interfaces.

[22] Lee J., Oh E.-T., Yoon H., Kim H., Park H.J., Kim C. (2016). Mesoporous Nanocontainer Gated by Stimuli-Responsive Peptide for Selective Triggering of Intracellular Drug Release. Nanoscale.

[23] Cai Y., Liang P., Tang Q., Yang X., Si W., Huang W., Zhang Q., Dong X. (2017). Diketopyrrolopyrrole-Triphenylamine Organic Nanoparticles as Multifunctional Reagents for Photoacoustic Imaging-Guided Photodynamic/Photothermal Synergistic Tumor Therapy. ACS Nano.

[24] Liu X., Zhang X., Zhu M., Lin G., Liu J., Zhou Z., Tian X., Pan Y. (2017). PEGylated Au@Pt Nanodendrites as Novel Theranostic Agents for Computed Tomography Imaging and Photothermal/Radiation Synergistic Therapy. ACS Appl. Mater. Interfaces.

[25] Ke W., Li J., Mohammed F., Wang Y., Tou K., Liu X., Wen P., Kinoh H., Anraku Y., Chen H. (2019). Therapeutic Polymersome Nanoreactors with Tumor-Specific Activable Cascade Reactions for Cooperative Cancer Therapy. ACS Nano.

[26] Li L., Yang Z., Fan W., He L., Cui C., Zou J., Tang W., Jacobson O., Wang Z., Niu G. (2020). In Situ Polymerized Hollow Mesoporous Organosilica Biocatalysis Nanoreactor for Enhancing ROS-Mediated Anticancer Therapy. Adv. Funct. Mater..

[27] Xue T., Xu C., Wang Y., Wang Y., Tian H., Zhang Y. (2019). Doxorubicin-Loaded Nanoscale Metal-Organic Framework for Tumor-Targeting Combined Chemotherapy and Chemodynamic Therapy. Biomater. Sci..

[28] Shen Z., Song J., Yung B.C., Zhou Z., Wu A., Chen X. (2018). Emerging Strategies of Cancer Therapy Based on Ferroptosis. Adv. Mater..

[29] Wang S., Li F., Qiao R., Hu X., Liao H., Chen L., Wu J., Wu H., Zhao M., Liu J. (2018). Arginine-Rich Manganese Silicate Nanobubbles as a Ferroptosis-Inducing Agent for Tumor-Targeted Theranostics. ACS Nano.

[30] Hao Y.N., Zhang W.X., Gao Y.R., Wei Y.N., Shu Y., Wang J.H. (2021). State-of-the-Art Advances of Copper-Based Nanostructures in the Enhancement of Chemodynamic Therapy. J. Mater. Chem. B.

[31] Duan X., He C., Kron S.J., Lin W. (2016). Nanoparticle Formulations of Cisplatin for Cancer Therapy. WIREs Nanomed. Nanobiotechnol..

[32] Kim H.J., Lee J.H., Kim S.J., Oh G.S., Moon H.D., Kwon K.B., Park C., Park B.H., Lee H.K., Chung S.Y. (2010). Roles of NADPH Oxidases in Cisplatin-Induced Reactive Oxygen Species Generation and Ototoxicity. J. Neurosci..

[33] Ma P., Xiao H., Yu C., Liu J., Cheng Z., Song H., Zhang X., Li C., Wang J., Gu Z. (2017). Enhanced Cisplatin Chemotherapy by Iron Oxide Nanocarrier-Mediated Generation of Highly Toxic Reactive Oxygen Species. Nano Lett..

[34] Chu C., Lyu X., Wang Z., Jin H., Lu S., Xing D., Hu X. (2020). Cocktail Polyprodrug Nanoparticles Concurrently Release Cisplatin and Peroxynitrite-Generating Nitric Oxide in Cisplatin-Resistant Cancers. Chem. Eng. J..

[35] Marques M.P.M., Gianolio D., Cibin G., Tomkinson J., Parker S.F., Valero R., Lopes R.P., Carvalho L.A.E.B. (2015). A Molecular View of Cisplatin’s Mode of Action: Interplay with DNA Bases and Acquired Resistance. Phys. Chem. Chem. Phys..

[36] Zhang W., Tung C.H. (2018). Redox-Responsive Cisplatin Nanogels for Anticancer Drug Delivery. Chem. Commun..

[37] Xiang H., You C., Liu W., Wang D., Chen Y., Dong C. (2021). Chemotherapy-Enabled/Augmented Cascade Catalytic Tumor-Oxidative Nanotherapy. Biomaterials.

[38] Zhang Q., Zhang P., Li S., Fu C., Ding C. (2019). Aggregation-Disaggregation-Switched Sensing Strategy for Copper(II) Ion and Histidine in Aqueous Solution and Living Cell Imaging. Dye. Pigment..

[39] Eldin M.M., Rahman S.A., Fawal G.F.E. (2020). Novel Immobilized Cu^2+^-Aminated Poly (methyl methacrylate) Grafted Cellophane Membranes for Affinity Separation of His-Tag Chitinase. Polym. Bull..

[40] Ueda E.K.M., Gout P.W., Morganti L. (2003). Current and Prospective Applications of Metal Ion-Protein Binding. J. Chromatogr. A.

[41] Chen Q., Manning C.D., Millar H., McCabe F.L., Ferrante C., Sharp C., Arruda L.S., Doshi P., Nakada M.T., Anderson G.M. (2008). CNTO 95, a Fully Human Anti αv Integrin Antibody, Inhibits Cell Signaling, Migration, Invasion, and Spontaneous Metastasis of Human Breast Cancer Cells. Clin. Exp. Metastasis.

[42] Bisanz K., Yu J., Edlund M., Spohn B., Hung M.C., Chung L.W.K., Hsieh C.L. (2005). Targeting ECM-Integrin Interaction with Liposome-Encapsulated Small Interfering RNAs Inhibits the Growth of Human Prostate Cancer in a Bone Xenograft Imaging Model. Mol. Ther..

[43] Cooper C.R., Chay C.H., Pienta K.J. (2002). The Role of αvβ3 in Prostate Cancer Progression. Neoplasia.

[44] Mas-Moruno C., Beck J.G., Doedens L., Frank A.O., Marinelli L., Cosconati S., Novellino E., Kessler H. (2011). Increasing αvβ3 Selectivity of the Anti-Angiogenic Drug Cilengitide by N-Methylation. Angew. Chem. Int. Ed..

[45] Zhang L., Shan X., Meng X., Gu T., Lu Q., Zhang J., Chen J., Jiang Q., Ning X. (2019). The First Integrins β3-Mediated Cellular and Nuclear Targeting Therapeutics for Prostate Cancer. Biomaterials.

[46] Zhang W., Tung C.H. (2018). Lysosome Enlargement Enhanced Photochemotherapy Using a Multifunctional Nanogel. ACS Appl. Mater. Interfaces.

[47] Zhang W., Zhe Z., Tung C.H. (2017). Beyond Chemotherapeutics: Cisplatin as a Temporary Buckle to Fabricate Drug-Loaded Nanogels. Chem. Commun..

[48] Parasuraman P., Antony A.P., Sharan A., Siddhardha B., Kasinathan K., Bahkali N.A., Dawoud T.M., Syed A. (2019). Antimicrobial photodynamic activity of toluidine blue encapsulated in mesoporous silica nanoparticles against *Pseudomonas aeruginosa* and *Staphylococcus aureus*. Biofouling.

[49] Anju V.T., Paramanantham P., Sruthil Lal S.B., Sharan A., Syed A., Bahkali N.A., Alsaedi M.H., Kaviyarasu K., Busi S. (2019). Antimicrobial photodynamic activity of toluidine blue-carbon nanotube conjugate against *Pseudomonas aeruginosa* and *Staphylococcus aureus*—Understanding the mechanism of action. Photodiagn. Photodyn. Ther..

[50] Parasuraman P., Anju V.T., Lal S.B., Sharan A., Busi S., Kaviyarasu K., Arshad M., Dawoud T.M.S., Syed A. (2019). Synthesis and antimicrobial photodynamic effect of methylene blue conjugated carbon nanotubes on *E. coli* and *S. aureus*. Photochem. Photobiol. Sci..

[51] Yuan P.X., Deng S.Y., Zheng C.Y., Cosnier S., Shan D. (2017). In Situ Formed Copper Nanoparticles Templated by TdT-Mediated DNA for Enhanced SPR Sensor-Based DNA Assay. Biosens. Bioelectron..

[52] Xu A., Li X., Ye S., Yin G., Zeng Q. (2011). Catalyzed Oxidative Degradation of Methylene Blue by in Situ Generated Cobalt (II)-Bicarbonate Complexes with Hydrogen Peroxide. Appl. Catal. B.

[53] Chen Y., Chen H., Guo L., He Q., Chen F., Zhou J., Feng J., Shi J. (2010). Hollow/Rattle-Type Mesoporous Nanostructures by a Structural Difference Based Selective Etching Strategy. ACS Nano.

[54] Sulkowski E. (1996). Immobilized Metal-ion Affinity Chromatography: Imidazole Proton Pump and Chromatographic Sequelae. I. Proton Pump. J. Mol. Recogn..

[55] Liu D., Liu M., Wan Y., Zhou X., Yang S., An L., Huang G., Tian Q. (2021). Remodeling endogenous H_2_S microenvironment in colon cancer to enhance chemodynamic therapy. Chem. Eng. J..

[56] Huang Z., Huang Y., Chen M., Chen J., Zeng Z., Xu X., Huang B., Luo Y., Xiao Z., Ding Y. (2020). Bone-targeted oxidative stress nanoamplifier for synergetic chemo/chemodynamic therapy of bone metastases through increasing generation and reducing elimination of ROS. Chem. Eng. J..

[57] Hao Y., Gao Y., Fan Y., Zhang C., Zhan M., Cao X., Shi X., Guo R. (2022). A tumor microenvironment-responsive poly (amidoamine) dendrimer nanoplatform for hypoxia-responsive chemo/chemodynamic therapy. J. Nanobiotechnol..

[58] Ren Z., Sun S., Sun R., Cui G., Hong L., Rao B., Li A., Yu Z., Kan Q., Mao Z. (2020). A Metal-Ppolyphenol-Coordinated Nanomedicine for Synergistic Cascade Cancer Chemotherapy and Chemodynamic Therapy. Adv. Mater..

[59] Ji X., Kong N., Wang J., Li W., Xiao Y., Gan S.T., Zhang Y., Li Y., Song X., Xiong Q. (2018). A Novel Top-Down Synthesis of Ultrathin 2D Boron Nanosheets for Multimodal Imaging-Guided Cancer Therapy. Adv. Mater..

[60] Vaghasiya K., Ray E., Sharma A., Katare O.P., Verma R.K. (2020). Matrix Metalloproteinase-Responsive Mesoporous Silica Nanoparticles Cloaked with Cleavable Protein for “Self-Actuating” On-Demand Controlled Drug Delivery for Cancer Therapy. ACS Appl. Bio Mater..

[61] Huang L., Liu M., Mao L., Xu D., Wan Q., Zeng G., Shi Y., Wen Y., Zhang X., Wei Y. (2017). Preparation and Controlled Drug Delivery Applications of Mesoporous Silica Polymer Nanocomposites Through the Visible Light Induced Surface-Initiated ATRP. Appl. Surf. Sci..

[62] Wu X., Wu Y., Ye H., Yu S., He C., Chen X. (2017). Interleukin-15 and Cisplatin Co-Encapsulated Thermosensitive Polypeptide Hydrogels for Combined Immuno-Chemotherapy. J. Control. Release.

