# Supplementary material for: Cu2+-Chelating Mesoporous Silica Nanoparticles for Synergistic Chemotherapy/Chemodynamic Therapy
Source: Pharmaceutics. 2022 Jun 4;14(6):1200. doi: 10.3390/pharmaceutics14061200 (PMC9229203; doi:10.3390/pharmaceutics14061200)
Supplement: Supplementary file 1 [file pharmaceutics-14-01200-s001.zip › pharmaceutics-1701388-supplementary.pdf]

Supporting Information

Cu^{2+}-chelating mesoporous silica nanoparticles for synergistic chemotherapy / chemodynamic therapy

Yanyan Zhang[1,†], Jiadong Lou[1,†], Gareth R. Williams[2], Yuhan Ye[1], Dandan Ren[1], Anhua Shi[4], Junzi Wu[4], Wenling Chen[3,*], Li-Min Zhu[1,*]

[1] College of Chemistry, Chemical Engineering and Biotechnology, Donghua University, Shanghai, 201620, P. R. China; zhangyy518@163.com (Y.Z.); 847603913@qq.com (J.L.); 1294878367@qq.com (Y.Y.); 2574125897@qq.com (D.R.)

[2] UCL School of Pharmacy, University College London, 29-39 Brunswick Square, London, WC1N 1AX, UK; g.williams@ucl.ac.uk (G.R.W.)

[3] School of Clinical Medicine, Yunnan University of Chinese Medicine, Kunming, Yunnan, 650500, P. R. China

[4] The Key Laboratory of Microcosmic Syndrome Differentiation, Education Department of Yunnan, Yunnan University of Chinese Medicine, Kunming, Yunnan, 650500, P. R. China; 2697349858@qq.com (A.S.); xnfz@ynutcm.edu.cn (J.W.)

* Correspondence: ynwenling09@126.com (W.C.); lzhu@dhu.edu.cn (L.-M.Z.)

[†]These authors contributed equally to this work.

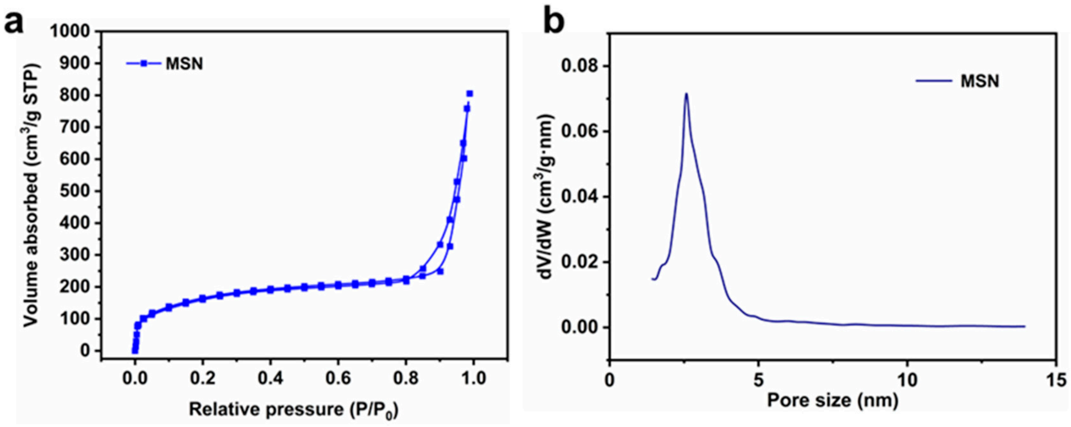

Figure S1. (a) N_2 absorption-desorption isotherms and (b) corresponding pore-size distribution of the MSNs.

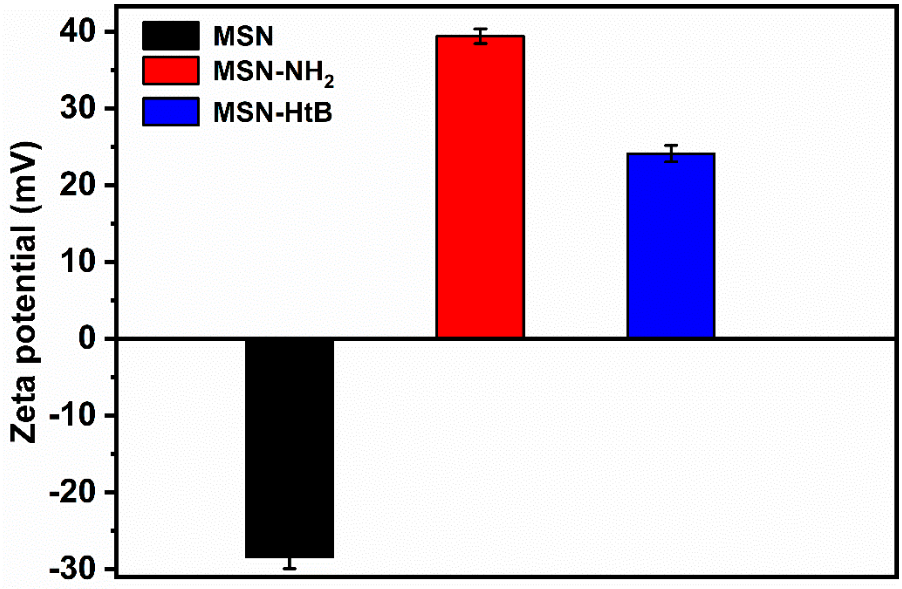

Figure S2. Zeta potential results of MSN, MSN-NH_2 and MSN-HtB.

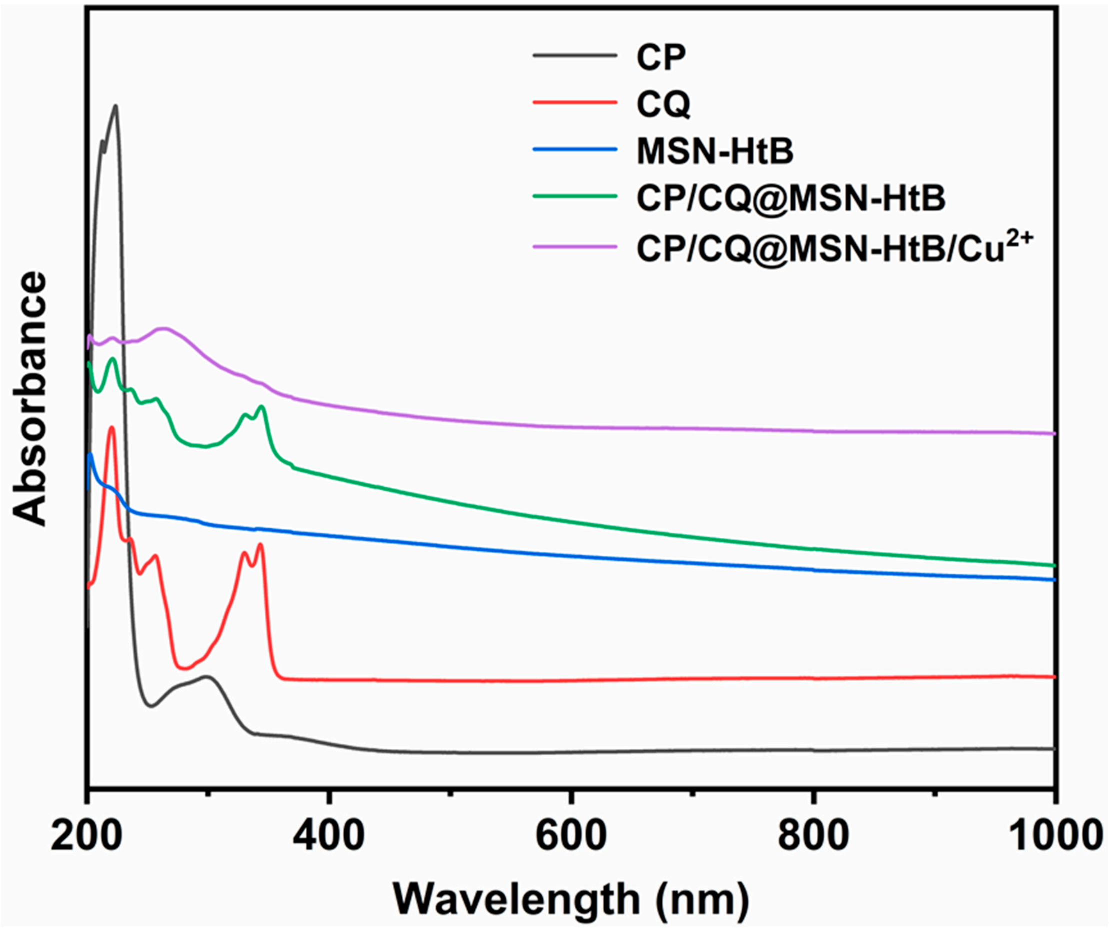

Figure S3. UV-Vis absorption spectra of free CP, CQ, MSN-HtB, CP/CQ@MSN-HtB and CP/CQ@MSN-HtB/Cu^{2+}.

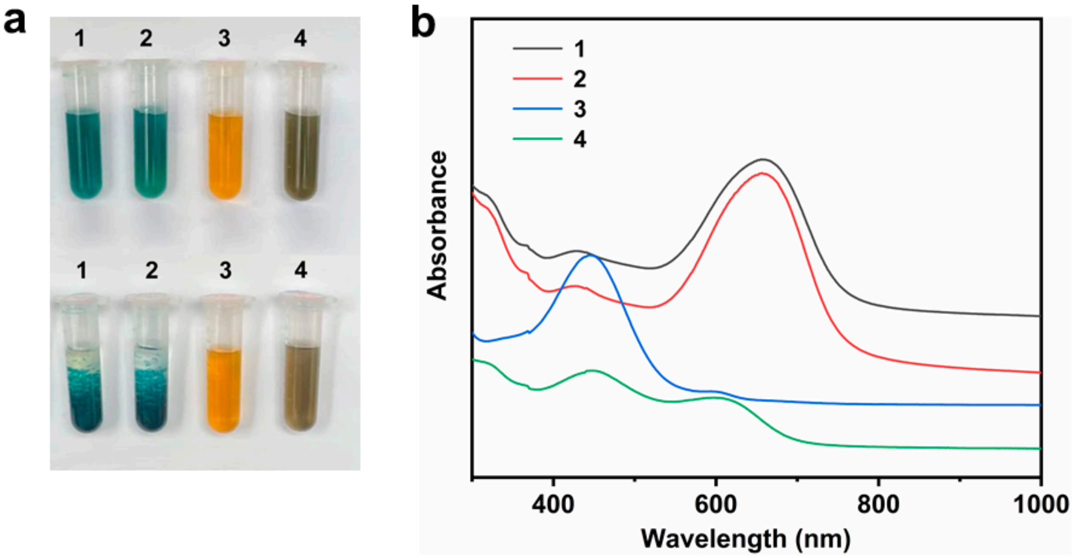

Figure S4. (a) Visual detection of chelation between Cu^{2+} and the His-tag by PV (top: immediately after the addition of PV; bottom: after standing for 15 min) and (b) the corresponding UV/vis spectra. Sample key: 1. MSN-HtB NPs; 2. HtB peptide; 3. control, without $CuSO_4$; 4. control, without HtB.

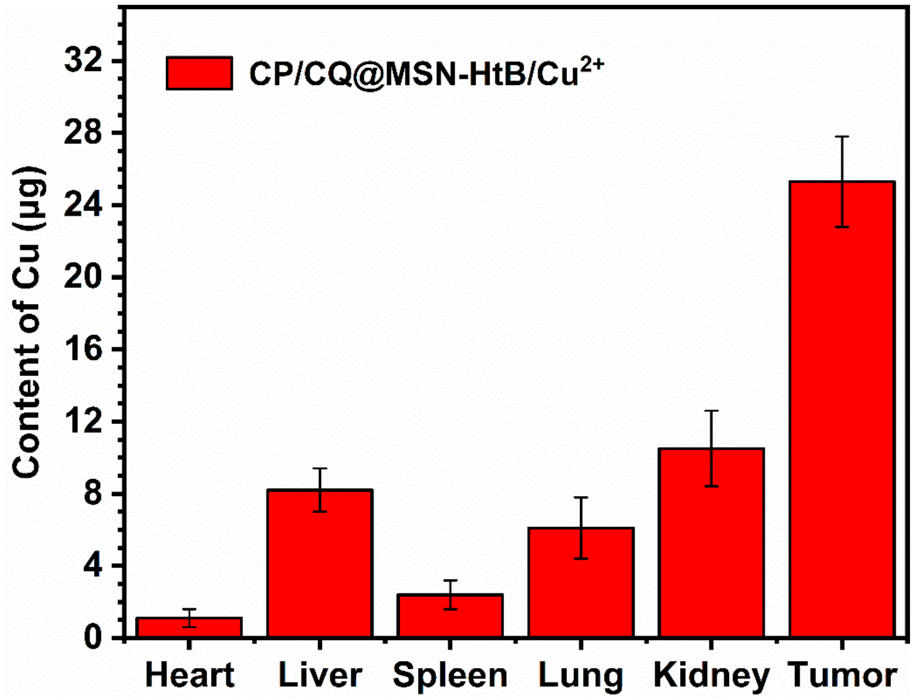

Figure S5. Content of Cu in the major organs and tumor after intravenous injection of CP/CQ@MSN-HtB/Cu^{2+} NPs.

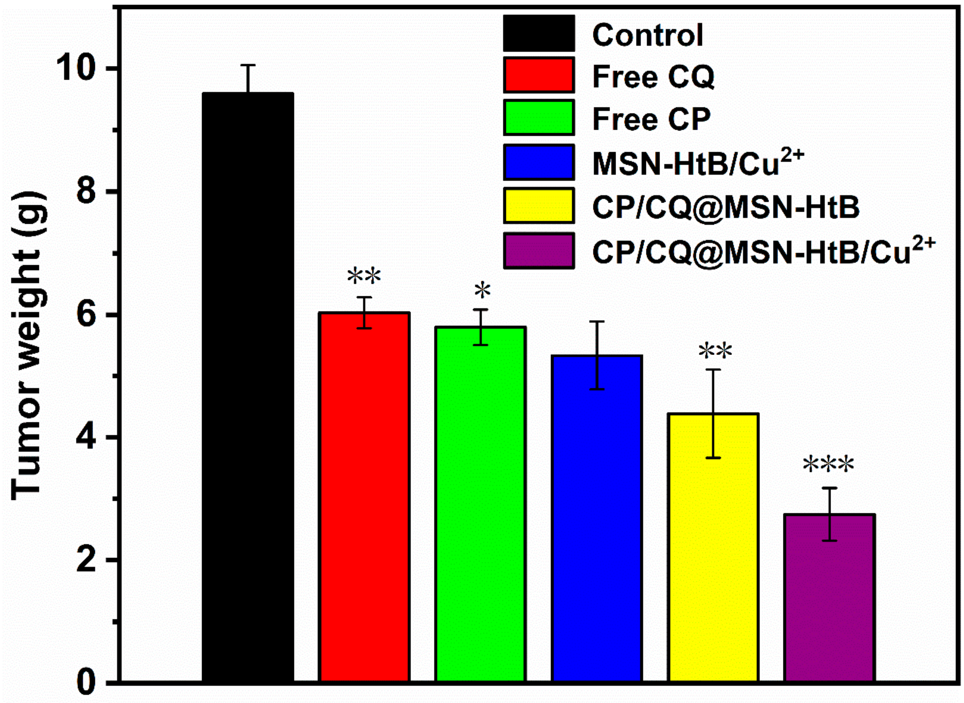

Figure S6. The tumor weights after the 12 day *in vivo* experimental period. *P < 0.05, **P < 0.01, ***P < 0.001 when compared to the control.

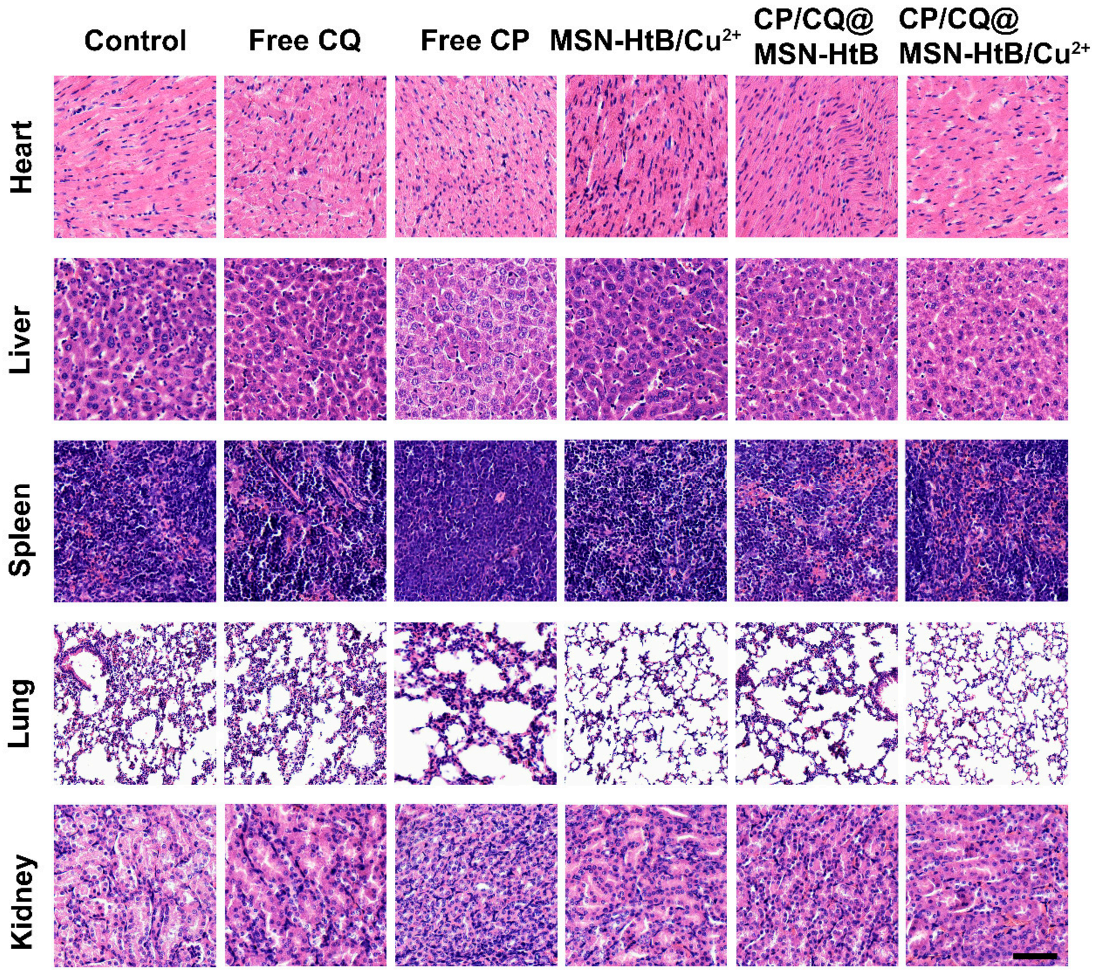

Figure S7. H&E staining of major organs from the control and experimental groups. All scale bars are 20 µm.
